# “Open science” meets commercial realities: a qualitative study of factors influencing sharing in synthetic biology research in Australia

**DOI:** 10.3389/fbioe.2025.1604509

**Published:** 2025-06-03

**Authors:** Alison McLennan, Sarah Maslen

**Affiliations:** Faculty of Business, Government and Law, University of Canberra, Canberra, Australia

**Keywords:** synthetic biology, sharing, intellectual property, open science, commercialization, openness, patent

## Abstract

This paper examines sharing of data and materials in synthetic biology research and the impact of intellectual property regulation and commercialization imperatives. Data-sharing, access to scientific knowledge, ownership of that knowledge and collaboration are critical issues in biotechnology research, as highlighted in the recent COVID-19 pandemic. We present a sociolegal investigation of drivers of sharing and hindrances to these activities in synthetic biology. This field has a particular emphasis on driving innovation through openness and sharing of the building blocks of research, as opposed to using intellectual property (IP) rights to limit access to these. We examine the perspectives and practices of synthetic biologists in both university and commercial settings, as well as commercialization professionals. We argue that synthetic biologists simultaneously manage two sets of imperatives. On the one hand, sharing is driven by cultural norms, pursuit of scientific progress and strategic benefits to the sharer. On the other, synthetic biologists need to protect their scientific careers, preserve the patentability of developments with commercial potential, and manage obligations to commercial partners and institutions. As their careers may not be purely academic or commercial, they need to appreciate the prerogatives of the particular “hat” that they are wearing on a given project, and also form judgments of commercial value, drawing on a distinction between fundamental and applied research.

## 1 Introduction

There is tension in synthetic biology around ownership and access to foundational tools and data. There are now many patents in synthetic biology, and the scale and complexity of the patent landscape is likely to increase as the field develops (McLennan, 2012, 2018). Alongside this patent landscape, there are also groups of synthetic biologists who are concerned about intellectual property (IP) being a hindrance to innovation in the field. Since the very early days of synthetic biology community, there have been strong themes of “openness”, “togetherness” and “freedom” (of access) (Endy, 2005; Boyle, 2008; Calvert and Frow, 2015; Mclennan, 2018; McLennan and Maslen, 2023; Torrance, 2017). As a way of addressing their concerns about IP, key synthetic biology institutions such as the International Genetically Engineered Machine competition and the BioBricks Foundation have designed their own communities and strategies for sharing foundational tools, information and materials (Boyle, 2008; Torrance, 2017). More recently, this philosophy has continued through projects such as the OpenPlant initiative (OpenPlant, 2018).

Synthetic biology’s emphasis on openness distinguishes it from other fields of biotechnology which have relied on patenting (Torrance, 2017). This makes it an important field in which to explore the impact of IP on innovation in scientific research. There is published literature indicating that some of these sharing initiatives are user generated solutions to concerns about IP hindrances (Boyle, 2008; Minssen and Wested, 2015; Mclennan, 2012, 2018). This means that the nature of the perceived IP problems is important to understand. However, there is a lack of empirical data about synthetic biologists’ experiences with IP problems, their strategies for managing these and their motivations to share intellectual and biophysical resources.

Accordingly, this paper investigates the nature of IP concerns and problems experienced by synthetic biologists, and their practices and motivations around sharing. Here, “sharing” describes the practice of giving information, data, or materials to another without receiving financial payment. As we will see, there may still be a benefit given to the sharer by the recipient. “Openness” here refers to a norm or a value that may underpin sharing interactions. “Collaboration” is a way of doing scientific research in which multiple research entities work together in a coordinated fashion, to complete a particular project or pursue a particular goal. This is likely to involve sharing activities.

We conducted interviews with scientists and the commercialization and legal personnel who support their activities to examine the extent to which IP concerns are a driver for sharing, and other ways in which IP impacts on the doing of synthetic biology research. We focus here on how practicing scientists navigate the tension between competing norms of openness and secrecy in the IP context, including motivations for sharing and hindrances to sharing. We show how synthetic biologists practice selective sharing guided by a sense of responsibility to advance the field and reap career benefits while also protecting their scientific credit and preserving obligations to partners and the patentability of developments with commercial potential. This selective sharing is guided by trust, reputation and relationships, along with seeking reciprocity in sharing interactions. It presents challenges in distinguishing between fundamental and applied research. As working scientists frequently find themselves involved in different kinds of research working for different employers, they develop an awareness of how their role on a given project must affect their sharing decisions and adjust their practices accordingly.

## 2 Sharing and “open” science

The “open” approach to the doing of science has long been held up as an ideal within the social study of science. Merton (1973) presented a vision of science as a social system in which there is a free exchange of scientific ideas, with the reward one of recognition. More recently scholars have critiqued these imaginings (Shapin, 2008). While scientists publish and present their work, they also engage in secrecy and adopt protective strategies (Mulkay and Edge, 1973; Mitroff, 1974; Knorr-Centina, 1999; Hackett, 2005). At least in universities, being first to publish scientific discoveries is professionally important (Hackett, 2005). Such pressure generates concern about one’s discoveries being “scooped”, with competing research teams sometimes going so far as to publish the findings of other groups that they accessed through open research websites (Marshall, 2002). Thus the doing of science can be characterised by tensions and ambiguities, as scientists hold contradictory norms as they go about their work (Hackett, 2005). They are locked in a set of relations in which they are one another’s audience and competition; their means to gain advantage and their greatest threat to achieving it.

Rather than seeing this issue as a case of openness versus secrecy, we can explore the dynamics and nuances surrounding these issues in the practice of science. This includes how these imperatives relate to the roles held by the scientist. Scholars have observed how scientists engage in “strategic information exchange” (Evans, 2010) with the goal of achieving scientific credit for their work. Sharing, or not sharing, in scientific work can manifest in different ways including presenting or remaining silent on findings in a research seminar (Cambrosio et al., 2004). Scientists may also choose to be open with their methods and materials through practices such as the timely upload of data into databases, or they may choose to withhold it (Hine, 2006).

As Levin and Leonelli (2017) explained, we see what researchers value not only in what they share, but in what they keep hidden. Their professional identities as working scientists are both independent and interdependent, requiring that they carve out their contributions while also seeking and benefiting from the intellectual stimulation and recognition that comes from “para-collaboration” (Hackett, 2005). Success can come from avoiding competition, practicing what Hackett (2005) termed “strategic reticence”, in which scientists may both share their techniques and keep them to themselves.

There are also a variety of reasons why researchers may not share. Outside of the academy, there is fierce protection of results and methods due to commercial sensitivity, IP considerations, and even national security (Resnik, 2006). Scientific discovery is but one part of a landscape in which it is commercialization of various services and products that are the primary concern. Such considerations of profit can lead to concealment of scientific discoveries (Evans, 2010). Further, commercialization of scientific research is increasingly expected within many scientific fields, including synthetic biology.

Scientists are also increasingly involved in large-scale collaborative research. These approaches tend to be deployed to respond to pressing health and environmental challenges (Love et al., 2021; Morrison-Smith et al., 2022). For example, scholars observed that during the COVID-19 pandemic, scientists demonstrated more open, collaborative approaches to research and deployed new digital tools for collaboration and dissemination of knowledge (Pipere and Mārtinsone, 2023). They rallied around mapping the virus, quickly sequencing it and putting this knowledge in the public domain via online platforms. This data sharing built on a culture of openness in genomic research, and forms part of the context in which vaccines were developed and deployed in record time (Monrad et al., 2021). However, these large-scale life sciences projects also create challenges in terms of managing and sharing knowledge amongst a collaborative group, deciding what will be shared and what will be will potentially be commercializable, as commercial realities cannot be ignored. In the case of Covid vaccines, complex patent disputes arose (see Aquino-Jarquin, 2022).

Within the synthetic biology domain, there are two perspectives on the role of IP in facilitating innovation (Kumar and Rai, 2006; Calvert, 2012; Nelson, 2014). In one approach, patents are being sought for various synthetic biology technologies. Patent rights are seen as essential to drive innovation and progress (see Nelson, 2014) due to the required investment for biotechnology research and getting innovations from the laboratory to the market. In this approach, synthetic biology technologies are seen as clearly the result of human intervention in nature, and so appropriate subject matter for patent protection (Mclennan, 2018). Patents for various applications of synthetic biology have already been granted in the US (Mclennan, 2017a, 2017b, 2018). Patent activity is also occurring in Australia, on a smaller scale (Gray et al., 2018).

In contrast, some synthetic biologists see IP as a potential hindrance to research in synthetic biology, at least when it is applied to restrict access to foundational, enabling technologies or “building blocks” (Calvert, 2012; Torrance, 2017). This is part of a broader discussion about the impact of patent laws on innovation and access to socially beneficial technologies (Butt et al., 2024). In synthetic biology, some scientists have chosen to make such things “open” or conditionally disclose such building blocks and knowledge about their use to promote innovation in the field (Calvert, 2012; Mclennan, 2018; Torrance, 2017). The emphasis on sharing and openness has been present in synthetic biology research since the early 2000s (Boyle, 2008; Calvert and Frow, 2015; Torrance, 2017; see Mclennan, 2018; McLennan and Maslen, 2023). This philosophy has been developed through key synthetic biology institutions such as the International Genetically Engineered Machine competition and the BioBricks Foundation. These groups have designed their own communities and tools for sharing foundational data, materials and tacit knowledge (Boyle, 2008; Torrance, 2017).

Sharing of data between communities of scientists can be likened to sharing within communities of innovators in other fields such as information and computer technologies. Indeed, some synthetic biologists have drawn inspiration from the user-generated solutions to copyright issues in that field (Minssen and Wested, 2015). Theory drawn mainly from these fields suggests that innovation can be enhanced if users share data in a cooperative, organised way within “innovation communities” (von Hippel, 2005).

In addition, studies of group management of environmental resources in “commons” suggests that sharing can enhance the return for each user (Ostrom, 1990). More recently, scholars have explored sharing in the age of “knowledge commons” (Hess and Ostrom, 2007; Strandburg et al., 2017). They have demonstrated that, like natural resources, “governance of intangible, intellectual resources too may be effective without recourse to traditional intellectual property” (Torrance, 2017). The diverging approaches to IP and openness in synthetic biology raise important questions about how to best promote innovation, whether the approaches can co-exist (Calvert, 2012), and to what extent the choice of some synthetic biologists to pursue sharing and emphasise openness indicates problems with the patent system.

While there has been significant discussion of IP, sharing and openness in synthetic biology in the science, law and social sciences literature, we have little empirical research into how this is playing out in practice. Previous empirical studies on sharing also focus on groups of scientists working in a single research context, i.e., conducting foundational research in a university setting, as academics working in industry-funded research programs for universities, or as scientists employed in commercial settings (Knorr-Centina, 1999; Hine, 2006). And yet careers in science are infrequently this clear cut with scientists researching in universities able to contribute to start-ups and apply for patents. Many scientists work in roles where goals include both practical applications of the science and contributions to the community working in the field. This raises questions about how scientists understand and navigate the sharing of materials and data both in relation to IP law and their own changing roles. We contribute to addressing this research gap in what follows, and explore the role of IP and commercial interests.

## 3 Materials and methods

### 3.1 Research design

The project was designed to investigate how the IP law and policy environment affects Australia’s synthetic biology industry. This project adopted a mixed methods approach including interviews, surveys, and analysis of laws and other documents to explore “law in the real world” as a basis for making recommendations for law or legal policy (Bell, 2016). The research questions included queries about the impact of patenting on innovation in synthetic biology in Australia (Mclennan and Maslen, 2023). The focus was on identifying issues that scientists or commercialization professionals are encountering in ensuring freedom to operate and securing appropriate licenses. The research questions also encompassed questions about sharing, in particular:

1. What, why and how do synthetic biologists share data, knowledge and materials in Australia? What motivations and hindrances are involved in these sharing practices?
2. What kind of sharing arrangements would be useful for Australian synthetic biologists?
3. How are synthetic biologists’ sharing practices affected by IP in Australia?
4. Do concerns about the impact of IP (patents) on progress in the field sometimes motivate synthetic biologists to share? What specific IP challenges or concerns are involved?
5. How will sharing arrangements interact with funding policies and agreements?

This paper focuses on the research findings on sharing as it was explored in interviews. The interviews involved the multiple stakeholders involved in and affected by the IP landscape for synthetic biology including scientists, technology transfer officers, and legal managers. Participants were asked about what sharing initiatives they would find useful, and any policy issues related to sharing, but more often than not participants instead spoke about the ways that they were already sharing, or reasons they would or would not share.

Interview questions were developed drawing on the literature about potential IP problems that may impact on innovation in synthetic biology discussed above in Section 2. Some questions were designed to help determine the presence or absence of patent-related phenomena, and their nature. The literature regarding innovation communities, and “commons” was also drawn on to generate questions regarding views on sharing and motivations and drivers for sharing. In asking these questions, general views were often elucidated regarding what should be patented and what should be freely available for use by scientists.

### 3.2 Research participants

Initially, purposive sampling was used to identify potential participants (see Creswell and Poth, 2018). The relevant potential participants were:

1. Scientists with a research focus in synthetic biology working in Australia.
2. Legal, commercialization and technology transfer professionals (collectively “commercialization professionals”) involved in the work of seeking IP, managing funding and collaboration agreements and other work related to commercialization of synthetic biology research in Australia.

Internet research and the primary author’s existing knowledge of scientists working in the field were used to identify potential participants. These individuals were invited to participate in an interview via an email from the primary author. Sometimes, this initial contact resulted in suggestions of other potential participants to contact. At the end of interviews, participants were also asked if there was anyone they would suggest as a potential participant. These suggestions were followed up and further recruitment occurred through this snowball sampling.

A total of twenty-two semi-structured interviews were conducted. Fifteen of the interviews were conducted with scientists working in synthetic biology in Australia. These scientists were working in universities and other research settings such as start-ups and larger commercial enterprises. A further seven interviews were conducted with commercialization professionals.

Interviews took place in 2021–2022 and were conducted by the first author, who is a legal scholar and also has a degree in biology. Human research ethics committee approval was granted for this research (by the Commonwealth Scientific and Industrial Research Organisation Social Science Human Research Ethics Committee and the University of Canberra Human Research Ethics Committee). Participants gave informed consent. Due to social distancing requirements, interviews generally took place over Microsoft Teams, with two interviews conducted in person. The interviews lasted around an hour on average. Interviews were audio recorded for analysis with the consent of participants.

### 3.3 Data analysis

Interviews were professionally transcribed. We adopted an analytic approach which involved both a thematic analysis (Ezzy, 2013) as well as the generation of vignettes. Thematic analysis supports an in-depth exploration of patterns in how participants perceive and make sense of their work environments and their implications for openness and sharing. The generation of vignettes is a complementary analytic approach, focusing on the development of a single story for a participant which brings into focus the particularities of a given vantage point. The data were coded manually using the affordances of word processing software as opposed to using a program like NVivo. This choice was made due to the smaller number of interviews, and due to the focus on the individual accounts of participants. The two authors thematically coded the data independently, and then both authors reviewed and refined the codes and themes for the analysis.

The thematic analysis presented in this paper was attentive to participants perspectives, practices, and experiences with respect to sharing, including views about culture of openness within the field, issues related to trust, instances in which sharing is straightforward, and instances that prompt protective strategies. The research questions were open-ended with respect to sharing practices and their conditions. The thematic analysis identified patterns in scientists’ perspectives on sharing related to their employment i.e., were they employed in a university setting, or within a company pursuing commercialization of a product. As readers will note, closer examination of this emerging theme revealed how it was the nature of the research, rather than strictly the employer, that appeared to affect sharing practice. This observation led to closer examination of researchers’ roles and funding arrangements, as well as the challenges in judging commercial value. Participants are referred to by a project code, a single letter assigned chronologically to maintain anonymity.

## 4 Findings

### 4.1 Synthetic biology’s culture of openness and contributing to the field

Openness in synthetic biology is built into the infrastructure of the field, with standardised tools publicly available, and online databases for sharing gene sequences and protein structures. It is also woven into the interactions of individual researchers and research teams who share tools and materials among themselves.

The scientists interviewed used a range of research tools and components, and were working on various materials including plants, seeds, plasmids, strains of microorganisms, cell lines and DNA parts. They shared and received physical materials, as well as information including genetic sequences, unpublished data, published data, and experimental protocols. Further, they shared and received know how and experience. For example, some had helped another scientist with data analysis or taught methods they had developed to other labs. Some had received know-how such as equipment recommendations.

Synthetic biologists see publication as a key strategy to share findings. This is true for all scientists we spoke to. However, it is a particular imperative for researchers in academic roles. Synthetic biologists seek to put their work into pre-print repositories and publish it in discipline journals. Pre-print repositories and journal publication take important steps towards sharing of information, though there were some participants who would like to see this taken further. Published research tells of what works; what did not work remains hidden. Keeping the failures hidden slows down innovation, as other researchers effectively waste time repeating experiments that others already know will fail. Participant B, a researcher in a university setting, explained:

It is really worrying how much is not actually available… having access to experiments that other people have done where it has not worked is just as useful… not having that just waste so much time.

In general, the scientists reported that other scientists were generous, and they were able to obtain what they needed through informal *ad hoc* requests or more structured communities of practice such as mailing lists of people working in a particular field. L explained:

If you’re trying to build on someone else’s research and that research is published, you can usually email them and ask any questions you have and they’re generally pretty good at responding and giving you whatever you want to know… and then often there’s things that are not included in the publication that are important to keep working on the topic… and they’ll often just tell you those things if you ask.

Several participants noted the importance of being well-networked for this purpose.

Typically, this culture of openness related to the view that this is important to the development of the field. Sharing is seen as speeding up the pace of scientific research. Further, Participant T (a scientist working outside the university setting) explained that where labs are united behind a particular goal to address “big questions”, this can promote sharing:

Because then it is not just about your group trying to be the first… It becomes a bit more about a common goal to advance a specific challenge… It makes people feel a bit more, inclined maybe is the best word.

Participant W (a university-based scientist) shared this view, focusing on how open, collaborative research could maximise research productivity. A project involving sharing between labs had meant that they had been able to achieve outcomes that could not have been achieved on their own. H saw sharing as having several benefits relating to efficiency and coordination, including avoiding duplication of effort and “being able to address higher-order issues”.

Others felt that sharing raw data sets would allow more minds to work on, and potentially solve, problems, as different analytical approaches may generate different insights. Participant A, another researcher in a university setting explained:

The same data can be analysed in multiple different ways to generate new insights… I only have one brain and two hands and if me putting it up, lets it utilise the 8 billion brains… then I see that as a net gain.

Several scientists explained that they might share a research tool or method partly to help the field develop and give a benefit to other researchers. For example, Participant M, a scientist working in research environment outside the university setting, worked in a team that had developed a method that they wanted to share with others in the field. This was seen as a way of contributing to its advancement.

However, the culture of contributing and sharing in synthetic biology is not the only factor at play here. Scientists are also conscious that there are strategic benefits they can obtain from sharing a basic method or tool. Scientists benefit from their method being widely used, because they can access a range of users’ experience, aiding in optimisation. Participant Q (who has worked both in applied scientific research and in commercialization roles) explained that they saw such practices as “like the open source sort of system… the more people that are in the pool that are using it, the more developments it will make”.

Further, while there is a culture of openness, not everything is shared in synthetic biology. Participant G (a scientist working in applied research) explained that there is an important distinction between sharing of “fundamental” and “applied” findings:

People are making these things available… fundamental properties of genes and proteins and things. I think what’s not being shared is when… they start to take those components and turn them into… applications and technologies.

This distinction raises questions about how scientists navigate these different research contexts, which we investigate in the following.

### 4.2 How a scientist’s role and funding relates to values around accessibility, ownership, transparency and benefit

While some synthetic biologists appeared to hold personal values or beliefs about accessibility of scientific knowledge and public benefit, decisions about what to share more commonly seemed to be underpinned by the context of the research. This context includes the scientist’s employer and the source of funding. Some views expressed by scientists have knowledge generation, as opposed to development of commercial products, as their driver. These tend to be scientists in university roles. For example, university scientist Participant A described their motivation:

What would I do if I found one (a genetic sequence) that improved protein production by 30% or something? Honestly I think I’d just publish it and not think twice about it. I have no motivation to make money off it. I’d rather just be out there in the public domain… My main motivation is just more around like transparency and accessibility, … or reproducibility more broadly.

While A considered that they would prioritise making data accessible over making money, they acknowledged that, as a junior researcher employed within a university, they do not need to grapple with commercial profitability. In this research context, this altruistic view is easier to adopt.

In scientific research, reproducibility is important, meaning the degree to which the results from an experiment can be repeated. Participant N, another researcher employed by a university, emphasized that data accessibility was important for ensuring reproducibility in biological research:

Reproducibility in research science requires other people to be able to scrutinise what you did and when you’re working with these kind of large sets of data that actually requires you to make available both the primary material, the primary data that you generated as well as the tools, the software that you used to analyse it with… It is about being transparent and being able to back up the claims that you’re making.

University scientist Participant A had also remarked on the importance of reproducibility, as discussed above.

Participant N (a university-based scientist) was reflective about their approach to sharing and access and the academic research contexts:

I think that open science within this kind of, you know, datacentric environment is incredibly important and people who have grown up academically within that environment are very passionate about data sharing and transparency in how data is being collected, and advocacy for equity of access and equity of contribution are very important topics in the field at the moment.

N continued to explain how they viewed some information as a fundamental resource:

Information about us as humans and the species around us, belongs in the public domain… because it is such a fundamental resource for understanding who we are and how we are within our various environments and communities and how we’ve been shaped by our past.

Some scientists, also working principally in university roles, discussed where the benefits from their research might flow. Participant U (a university-based scientist) was concerned that relatively few people would benefit from a patent compared to the people who could benefit from the research. Participant R (a scientist who has worked in both university research and other research settings) was concerned about “making sure things do remain open for benefit”. They thought that more research tools should be free to be used, and only “really, really smart” developments made with those tools should be protected.

For some participants, openness, or careful consideration of the scope of patent claims, was also important because of the risk of negatively influencing public perceptions of the field if there were large monopolies. These participants referred to earlier GM technologies where very broad monopoly rights were held by large biotechnology companies and there was public backlash relating to this. Participant R (a scientist who has worked in both university research and other research settings) was concerned about

This sense that it is all going to be controlled by large multinational big companies is not appealing to a lot of people… that’s where–what happened with Monsanto and Roundup and, you know, where the whole GMO technology really got–came unstuck. So I think we’ve got to be careful for that… IP is there to encourage innovation, not stifle it.

Participant M, who has worked primarily in industry and applied research, shared a similar concern. They thought it was important that patents not be used in a way that meant overly large monopolies damaged public perceptions and social license relating to synthetic biology.

There were some participants who emphasized the necessity of IP protections. This typically related to the positions that they hold. Scientists working in non-academic settings (or with commercial funding) and those working in commercialization roles do not have scope to see accessibility as a key goal. They consider that investment is incompatible with open access. For example, Participant C, a commercialization professional in a university, observed that scientists in their organization shared a lot more, and gained a lot more from sharing, than scientists in other organizations where there was more pressure to commercialize.

Interestingly, some of the scientists were able to articulate how their perspectives on and practices of sharing are shaped by the research context, rather than being based on intransigent personal values. This means that it is possible for one scientist to vary the priorities in their decision-making process based on the nature of their current role. For example, Participant S, a scientist working in a university role and also in a startup, stated: *“it is almost like I need to do two interviews because I kind of have a split personality”*. Participant K, a university scientist who has also been involved in a startup company, similarly expressed:

I think there’s a lot of fabulous synthetic biology companies out there that are accelerating methodology for being able to build things faster and quicker and more complex systems. And they’ve got, of course, the IP associated with those processes. Do I have a problem with them doing that? Possibly no, because I know the degree of R&D and cost and money and time that has gone into building the technologies that they have built.

K acknowledged this perspective relates to K’s own role: “that’s the commercial side of it. If I was an academic I’d say, yeah, it should all be free… So it depends where you sit as to what your views are.” They can wear different “hats” in relation to openness and access to scientific knowledge in synthetic biology at different times.

The participants who acknowledged how the patent system may advance synthetic biology felt strongly about this view. Some suggested that those scientists who supported open access were naive about the level of work involved in moving a discovery from the R&D stage to a commercial application. The idea here is that IP is needed to protect patent holders and make it attractive for companies to invest in innovation. Participant D, a technology transfer professional in a University, explained:

The thing about the patenting system is it is a trade-off, right?… Incentivise the people who invest time and resources that might create the IP… (balanced) with the rights.

The involvement of a commercial partner or a commercial source of funding is a key factor in deciding what can be shared, as these arrangements bring with them strict requirements for confidentiality. Several scientists mentioned the relevance of this funding context. For example, Participant R, a scientist who has worked in both university research and other research settings, explained “we would not share anything in a commercial project. We would be contractually unable to do that without approval”. In relation to sharing, Participant V (a scientist in research outside the university setting) said “I think generally people want to do it, but there are some restraints that make it difficult and, if it is a commercial project, obviously, you cannot do that”. By contrast, some scientists pointed out that some public funders have requirements to put information in the public domain.

### 4.3 The problem of judging commercial value

Scientists are more trepidatious about sharing if their research has (or may have) commercial value, even those employed in academic settings. It is easy to say you support sharing when you do not have something of immediate commercial value to give away. The choice where there is potential commercial value is a truer test of the motivations and beliefs of scientists. Participant L, a university scientist, explained that in assessing whether to share, they would make a judgment about the value, holding off where they could commercialize something:

It would depend how commercially relevant I thought the research was. So if I had plans to commercialize it myself and I thought it was really valuable and that it had not necessarily had to be part of a publication, or a public disclosure, and I thought that the group that wanted to have that information would also try to commercialize it, then I probably would not share it.

Here L is referring to the requirement that for an invention to considered novel, the innovation must not have been publicly disclosed prior to the patent application (unless an application is filed within 12 months of the disclosure). In Australia, this requirement is found in the Patents Act 1990 (Cth) s 18, 24 and Patents Regulations 1991 (Cth) reg 2.2. As Participant J (a technology transfer professional working on commercialization of university research) explained, this decision has to be made first and then “as soon as we’ve filed a patent on something the researchers are free to go and publish”.

This was a key factor for scientists in determining what would and would not be shared, and the extent of disclosure of information through conferences, publications, and repositories. University scientist Participant P highlighted that this was one of the two main reasons for hesitating about sharing information, with the other being the possibility of ideas being stolen (the latter concern is discussed further below). Participant S, a university scientist who was also involved in a start-up, also mentioned the same two concerns. Participant P spoke of the strategic decisions involved in deciding what level of detail to present at conferences, because of the possibility that this information could find its way into the public domain and become discoverable later as part of a patent examination process.

Deciding whether a patent will be sought involves considering the potential for commercialization, and whether competitive advantage will be lost by sharing. For example, university scientist Participant L described the decision as to whether to share as being about “my estimation of their capacity to compete with me for commercialization”. By contrast, “if I thought the research was fundamentally academic and far from commercialization, I would always just share whatever they wanted”.

However, there were differing views on how easily commercial value can be judged. Participant K (a scientist who has worked primarily in universities but also in a startup) spoke of the difference between basic research “for pure discovery” and research that is “tailored towards building something that can… solve a problem”. For K the decision is clear, and made early on, with respect to commercial value and what that means in terms of behavior.

The research has to divide very early on, in a particular project, to either chase one or the other. It would be strange to start a project and midway through it discover that it’d have some commercial outcomes.

K had worked on a project where there was a clear decision point where research changed direction from pure research to research for a commercial outcome. They thought that “at some point in any research project, I think you make that conscious decision that the next R&D steps are to go off into a commercial side step or stay on a basic research side and share information”. University scientist Participant N also spoke about distinction between “fundamental” and commercial research and the deliberate decisions that would need to be made for research to take a commercial pathway.

Some scientists thought that it was possible to have two tiers of material or data, based on these distinctions. University scientist Participant L said that sometimes the distinction was easy. They gave the example of a project where a platform technology had been made freely available. If it was used by a research group to make something valuable, then that group would be able to own the IP and commercialize their development. University scientist Participant N thought that “a tiered approach could work as long as there is a clear understanding of when something no longer becomes commercially sensitive and can then be shared”.

Some participants drew a distinction between sharing of basic components or tools and innovations developed from these. They used this to delineate what should be shared and what should be kept secret. For example, Participant F (a person who has worked both in scientific research and in commercialization) thought that having communities in synthetic biology where participants shared genetic components without seeking patent protection would be “probably a very good way of accelerating innovation”. However, F thought that it was important that patent protection could be sought for potentially valuable inventions developed with these open access components.

This distinction between fundamental and applied things was a strong theme in the data. Participant G (a scientist working in applied research) thought that the distinction could be drawn between fundamental knowledge that is publicly available, and uses of the knowledge in a targeted way. They gave the example of the CRISPR technology, where the process of gene editing is in the public domain but if a researcher used it to make a cell strain that makes a particular chemical, they would keep that secret: “You only need that information if you want to compete with that company”.

Other participants gave further examples of this distinction in practice. For example, there are times when sharing occurs but there are conditions on use of what is being shared. Participant M (who has worked primarily in industry and applied research) had a research method their team had developed. They envisaged making this available to the research community, helping others to use the method, and thereby making a contribution to the field. But if M’s team were working with a commercial partner to use that method for a specific application, there would be secrecy around these details. In these cases where scientists sought to share a method or tool widely and benefit from users’ results, the usage is only “open” for (basic) research purposes. That is, users would need a license for commercial (applied) use of the method. Q explained “we want everyone to be using it as a research tool, but we, of course, do not want to give away commercial rights to anyone straightaway because some of these–someone may make some fantastic product with it”. V explained that a license would be needed for commercialization of an innovation made with the shared tool. In a similar vein, Participant K (a scientist who has worked primarily in universities but also in a startup) mentioned that a lot of the core technology they use is in the public domain, but they protect information about the improvements they have made to that core technology. For example, they would not disclose information about optimising a biological device.

Some scientists spoke about being unclear on whether their work had commercial value and so what that means for sharing. Participant T (a scientist working outside the university setting) thought that distinguishing between precompetitive data and data that might be commercially valuable can be tricky. They said that to a certain extent the distinction can be drawn, but in some projects they’re not sure whether there will be a commercial output at the end.

Technology transfer professionals also considered this a complex judgement. Participant Q (a scientist who has worked both in scientific research and in commercialization) stated that “I think that must be very difficult to determine what you try to hang onto it what you do not… To work out what sort of data would be foundational versus what would be commercially valuable”. Participant J, a technology transfer professional, expressed this view:

It is just sometimes you do not realise something has commercial value until after the fact… I think that would be difficult to answer and that’s where some of these things could start to unravel… the more fundamental the research is, the easier it would be to argue it is pre-competitive. But I’m not sure it would be easy to draw a line.

Participant H, also a technology transfer professional, noted issues in judging commercialization potential, and so the decision to share or not share:

I think you can draw it at a particular point in time for a particular purpose… That’s one of the things we struggle with. Like, it is sort of like it could have value. And you’re like, oh well, do you never share it because it could or are you–is the benefit of sharing it now potentially greater or–the benefit of sharing it now is enough for us to be comfortable and to be able to justify sharing?

Participant C, a commercialization professional working with a university, noted the complexity of this distinction, because “the data is not valuable on its own”; the context is important. They explained that “what someone might not be able to get any commercial value out of, another party may, because of their connections or their expertise”. Participant D, another commercialization professional working with a university, thought that sometimes scientists were wrong when they judged material to not have commercial value.

This issue of judging commercial value is heightened in the context of universities that are increasingly interested in the commercialization of research findings. Participant C explained:

The landscape is definitely changing… Universities and research organizations have, over the years, become more protective, less willing to share and more likely to want to negotiate ownership or royalties.

This is a pattern across the sector due to financial pressures on universities who seek alternative funding streams. Participant F (a scientist who has worked both in scientific research and in commercialization) observed a change in culture as research organizations have moved towards greater commercialization to compensate for reduced public funding:

The communities have been traditionally very open and you would find that conferences… They’d have lots of discussions with other scientists… And then, of course, as public research agencies started to protect IP more, mainly because of reduced government resources into their programs, they need to capture value through commercial arrangements… Then you start to see a bit less discussion around things and people being guarded, people wanting to be first to discover so they can get a patent so their star would rise.

This shows a tension between wanting to share as part of the traditional research culture, and having to maintain secrecy due to modern commercial realities. This tension had been experienced by Participant K, a scientist who had worked in both academic and commercial research. K’s commercialization office wanted the research group to “say nothing” until a particular point in the patenting process, but “we cannot do that as academics”. The university’s commercialization policies discouraged sharing, but K also needed to publish and present their work. University-based scientist Participant L also mentioned this tension in the context of competing policies that would apply to a research group’s decisions. L explained

There would be policies associated with publication and academic conduct, like that want you to freely share everything and then there would be tech transfer and IP policies that want you to share nothing. And it is basically up to us, where want to sit on that continuum.

In relation to policies encouraging sharing, L may be referring to the requirements to provide data when publishing research in scientific journals, and the policies of some public funders regarding putting information into the public domain. The competing policies regarding commercialization may be those of the employer.

Some of the university scientists interviewed dealt with university policies by ignoring or bypassing commercialization offices. K explained:

We now as a group very carefully look at what we can and cannot disclose. We also have the commercialization office with the patent, they’ve just shut us down. They do not want us to say absolutely anything. Now I find that going to the extreme. So we do tell them what we’d like to do, and they of course say, no, well, you can’t do that, and then we just do what we feel is appropriate anyway.

Along a similar line, university scientist Participant U said “I’m not going to tell my university tech transfer office that I’m going to put some plasmids on Addgene because I’ve made the assessment myself that these are not commercially relevant.” Another scientist mentioned that “We can do whatever we want, at our discretion”. University scientist Participant W thought that most academics would not even be aware of university policies in this area. Accordingly, university policies do not seem to be a key factor preventing scientists from sharing. Outside this sector, organizational policies and priorities were seen as having a greater impact on sharing, they were “unavoidable”.

### 4.4 IP, sharing and trust

The scientists we spoke with emphasized their relationships with other researchers in making sharing decisions, irrespective of their employers and funding arrangements. Participant W, a university scientist, described a decision process involved in sharing, based on who is asking:

There’s a decision tree in my mind with any conversation… who are they, how well do I know them, how well do I trust them and what do I need to tell them to achieve whatever our goal is… There’s always an internal debate of, what should we actually say to this person?… What do they need to know? How much do we trust them?

In one case, W’s team needed to grapple with how much information to provide to a potential funder. They did not want to lose competitive advantage. They settled on enough to prove the concept, but stopped short of giving some information. W reflected: *“I think that was the sweet spot and then they got the concept. They realised it worked but we did not give away everything that we’ve done.”* With a research collaborator, sharing would be less restricted.

Again, the work context is an important factor here, with sharing more appropriate in fundamental research in universities, as opposed to commercial development in companies. However, the choice to share is still based on trust. Participant K (a scientist who has worked primarily in universities but also in a startup) explained:

I find the community very generous. But that’s… with people just in basic R&D not commercialization areas… If I’ve wanted… a strain from overseas, people are more than happy to ship them… So there is good sharing in the community… but a lot of it is based on reputation and relationships… And because it is a small world and if you upset someone, people will know.

Participant T (a scientist working in applied research) said that they could sometimes draw a distinction between commercially valuable information and non-commercially valuable information. In both cases, they would require trust to share. They indicated that they may share even potentially commercially valuable information if they trusted the person, but could be unsure without first building the relationship:

I think you’re always a little bit reticent at first, right, until you know them or have at least met them and had a chat… and people’s reputation in an area can influence that too… It is a smallish community and people talk… There has to be that element of trust, that you think they’re not going to just walk off with it, develop it, commercialize it themselves and not acknowledge you or include you.

The importance of trust comes from a place where some scientists had experienced, or witnessed, violations of trust where a scientist or team had shared their ideas or preliminary research findings only to have them “stolen” by another team. University scientist Participant W was concerned about sharing at conferences in this context:

What we’ve learnt from bitter experience, is not to talk about anything cool and interesting… I’m a big fan of collaboration, but tempered by experience of not everyone collaborates in good faith or can be trusted.

This issue of trust is not only an issue of IP and commercialization opportunity, but also of career impacts. These can be particularly acute for junior scientists. Participant W explained that they guide students to present work at conferences that is not easily stolen or “scooped”. University scientist Participant L also said they were careful when dealing with a “power differential”.

A critical part of the trust equation is that the researcher who is sharing needs to benefit from this sharing; it is not entirely altruistic. When the scientists say their trust is violated, what they mean is that the reputational benefits that they would have accrued from their discoveries have been taken from them. Participant K (a scientist who has worked primarily in universities but also in a startup) expressed this in terms of there needing to be some kind of a “transaction”, some kind of reciprocity, to make the sharing okay. In academic circles, that is typically having your name on a paper. K explained that if this kind of reciprocity does not happen then there is more of a sense that payment and a Material Transfer Agreement (MTA) is needed.

I used to give the material to physicists all around the world, and they’d do these amazing experiments and I’d get my name on the paper, and that was payment enough for me… And then it got to the stage that my material was being handed to other groups and I was not being acknowledged… And that I found very upsetting. And so that’s when we took that to a commercial stage of, okay, if I cannot get benefits from having authorship on a paper, then I will sell my material and be reimbursed in that way… So there is a transaction of sorts that has to go on.

Other participants referred to having own papers cited or being attributed for their contribution. For example, where Participant V (a scientist working in applied research)’s group taught their method to other labs, they would expect their paper to be referenced in that lab’s related publications.

### 4.5 Sharing by not sharing

There is evidence of tactics to avoid sharing, while maintaining the appearance of sharing in line with the broader culture of the field. Journals require statements saying that materials are “openly available”. This forces scientists who are publishing to share in some way, such as through uploading their data to an online repository. For example, to publish in the prestigious Nature journals “authors are required to make materials, data, code, and associated protocols promptly available to readers without undue qualifications” (Nature Portfolio, 2025). This policy aims to promote reproducibility of results and facilitate research progress.

However, there are various reasons why a researcher may not want to share data outside this publication context. They may not trust the person asking. Research groups are ultimately competing for funding too. Participant L, a university-based scientist who had also worked on startup companies, explained how MTAs can be a device to maintain the appearance of participating in the culture sharing, while actually blocking access:

Some MTAs are made with the explicit purpose of never being signed by anyone because they’re too unreasonable… It is a tool that researchers use to make the appearance that they’re sharing their materials from a publication, but then in reality they never actually have to share them because the MTA is too absurd, no-one would sign it.

This shows that some synthetic biologists have developed a strategy to manage competing imperatives of openness and secrecy. They need to publish to maintain their academic career, but they also limit what they share to maintain their competitive advantage.

## 5 Discussion

Synthetic biologists need to navigate tensions between openness and secrecy in their practice, whether in university or in commercial settings. Our research explores not only formal sharing through publication, but also informal sharing. Scholars have noted the importance of such informal sharing of “methods, materials, and early manuscripts” as a form of communication between scientists (Evans, 2010; Levin et al., 2016). Our research explores in-depth how synthetic biologists go about making these decisions.

Scientists we spoke to were concerned to promote reproducibility and transparency through sharing. This reflects concerns that have been identified in discussions around open data sharing in biotechnology research more generally (Breznau, 2021). In our study, sharing data so that it could be verified and examined by others was seen as a way of demonstrating the credibility of the results being claimed to other scientists. That is, it is part of the process in which “scientists seek to assist the translation of individual claims into collectively credible knowledge” (Shapin, 2015).

The published conversation among synthetic biologists engaged in sharing initiatives emphasizes that openness is important because the accessibility of fundamental building blocks is critical to innovation in the field (Torrance, 2017). These building blocks are “the means of creation for user innovators” (Hilgartner, 2012). For example, proponents of Open Plant have stated that “IP practices and restrictive licensing threaten to restrict innovation as the scale of DNA systems increases” (OpenPlant, 2018.). They see openness as “necessary for innovation and equitable access to the new biotechnologies that will underpin future sustainable practices and the global bioeconomy” (OpenPlant, 2018). These discussions in synthetic biology are part of a broader discussion about whether IP achieves its objective of promoting innovation. As scholars have noted, there is disagreement on this issue (Hilgartner, 2012).

In ethical discussion around synthetic biology, access to and commercialization of knowledge are also regarded as important issues (Kurtoğlu et al., 2024). As Kurtoglu et al. point out, there are significant concerns about “the commercialization of the information produced and how it can be used for the public good”.

Our empirical work echoed some of the sentiments above around IP, ownership and access, but reveals more nuance and other key drivers for sharing. We highlighted how the navigation of particular circumstances requires that scientists to think within a given role and its prerogatives, or within the goals of the funder, which can change from one project to the next. These findings align with work by scholars such as Levin and Leonelli (2017), who demonstrated that “openness is inherently positional and relational and is subject to dramatic qualitative shifts depending on the characteristics of the locations involved or the personal relationships.”

The participants who were more concerned about openness and access tended to be engaged in “basic” research within universities, while those who saw patents as important for innovation tended to be engaged in research outside universities or were employed in technology transfer roles. Among university researchers, there were some who thought that some information and research tools should be openly accessible, as they were fundamental resources. There was also concern about whether patent monopolies were a good way to obtain benefit from the research. However, university researchers were not primarily or expressly driven to share by concerns about the impact of IP on innovation in the field. Instead, the scientists shared because their research field has a culture of openness (as has also been argued by Calvert, 2012; Torrance, 2017), they saw sharing as a way of promoting progress in the field, and they had a sense of individual career benefits in the form of publications and citations. In the commercial research context and among the commercialization professionals, IP was not generally seen as a block to innovation. IP was seen as an important part of the process of getting research to market. Participants pointed to the high cost of this process and the need to recoup investment. This meant that decisions as to whether to share, what to share and when were influenced by decisions about seeking patents.

However, our data show that the lines between academic and commercial research are not firm. Scholars have described how modern biotechnology has become an applied, commercial enterprise (see, for example, Evans, 2010; Torrance, 2017). This includes through developments in IP law and legislation that has allowed university commercialisation of research (Evans, 2010). Our findings support these observations, with participants pointing to reduced government funding and greater emphasis on patenting of university research.

Commercial pressures on university researchers mean that they often have to manage both the responsibility and culture of openness and the demands of their contractual arrangements with commercial funders. Scientists can be simultaneously involved in fundamental research in a university while also contributing to a startup. As has been found elsewhere, science is less open when it is conducted in partnership with industry (Evans, 2010; Borycz et al., 2023; Liu et al., 2024). Our data show that commercialization potential, the ability to attract commercial funding and the ability to pursue patents are all important considerations in university research which affect the decisions of university scientists and must be managed alongside the culture and responsibility of sharing in synthetic biology. That is, concerns about the impact of IP on innovation may not be key drivers for sharing activities in our study, but IP and commercialization potential are often key reasons *not* to share.

Practising synthetic biologists navigate these tensions through selective sharing, meaning carefully choosing when, what and with whom to share. This selective sharing is guided by trust. Scientists in this study consider the strength of their relationship with the potential recipient and the recipient’s reputation in the field before engaging in informal sharing. They pointed to situations where sharing resulted in ideas being stolen and where appropriate attribution was not given. These issues were particularly concerning for junior scientists and those supporting their careers. Our data showed that despite the general culture of openness, and their concern to advance the field, synthetic biologists will not engage in sharing with groups they regard as unscrupulous. These findings align with the work of scholars such as Levin et al., which has shown how scientists had to balance the importance of making data accessible to others with the need to protect scientific credit (Levin et al., 2016). In our data, the importance of trust and relationships meant that the academic scientists interviewed generally did not consider contracts to be an important tool in relation to sharing. Outside academic research, some scientists thought it was important to have both trust and a contract.

Scientists also navigate these tensions by seeking reciprocity in sharing activities. That is, sharing is not necessarily altruistic. They expect a benefit to the sharer in the form of attribution, co-authorship, citation (all forms of scientific credit), useful user knowledge, or a financial benefit. This is not unique to biological research. The importance of self-interest and reciprocity as factors impacting on sharing decisions has also been observed in other areas of science such as astronomy (Liu et al., 2024).

Critically, scientists also navigate their decisions about how to balance openness and secrecy by distinguishing between “fundamental” or “basic” and “applied” research. They share fundamental knowledge, data and research tools. They do not share “applied” information, that with commercial potential or relating to a commercial project. They do not share applied materials, such as engineered organisms that produce a particular product of interest for a commercial application. They do not share the things that they consider the most valuable (Levin and Leonelli, 2017). In our data, this was knowledge about the optimal way of doing something for a particular purpose. Further, our participants had ways of limiting the extent of sharing. When a tool is shared for “research use”, they may require the user to seek a license if they use it for commercial purposes.

However, the distinction between what is basic research or enabling tools and what is commercially valuable is not clear cut. As with the distinction between academic and commercial research, it is becoming increasingly important while simultaneously more fraught, with the general move to greater commercialization of university research and the multiple work contexts that scientists can be engaged with simultaneously. While some synthetic biologists in our study seemed to take the distinction for granted, commecialization and technology transfer professionals tended to consider this a difficult line to draw. Synthetic biology is a field driven by applications. As interviewees explained, there are few synthetic biology efforts that do not have a specific application in mind. This makes distinguishing between “basic” and “applied” research more complex in this field.

Nonetheless, the distinction our participants drew in relation to foundational and commercially valuable technologies does reflect a distinction drawn in some large-scale sharing initiatives in synthetic biology. For example, the OpenPlant project takes a two-tiered approach to sharing and IP. As its proponents explain, “While freedom to operate is necessary for foundational technologies, the commercial applications and products that will be built upon these foundational technologies require investment in development, production and distribution for which IP protection is usually necessary” (Kahl and Molloy, 2018). Accordingly, they have created a two-tier approach in which “low-level technologies with little commercial value in isolation or with high potential to spur innovation are made available openly while high-value applications may be patented or otherwise protected (Kahl and Molloy, 2018). This suggests that the proponents of Open Plant are more aligned with the position of our participants who thought this distinction could be readily drawn.

The features of selective, informal sharing that we have described mean that the scientists in this study are often not engaging in “free revealing” as described in the literature about innovation in innovation communities (von Hippel, 2005). This is where “intellectual property rights to that information are voluntarily given up by that innovator and all parties are given equal access to it—the information becomes a public good” (Von Hippel, 2005). While some sharing noted in this study can fall into this category (putting information on some public repositories, publishing in a journal), often the scientists are engaging in more limited strategic disclosure only to specific individuals or lab groups. Nonetheless, we can see that some theoretical benefits of free revealing will still be obtained. For example, the recipient’s use of the shared method or information may yield improvements or “debugging” that can be useful to the sharer (von Hippel, 2005). This was a key reason for sharing mentioned in this study.

The tension between openness and secrecy that we have highlighted can be seen in competing policies and requirements that must be navigated as scientists make decisions about selective sharing. As we have seen, some public funders require the disclosure of data into the public domain and academic career imperatives require publication. Scholars have noted a global trend of government bodies adopting policies to promote openness in science (Levin et al., 2016). In this context, multiple government open access policies can apply to scientific research, which can create conflicting and confusing requirements (Levin et al., 2016). Our data highlighted a further complication in terms of applicable policies that scientists must navigate. With increased university commercialization, commercialization offices are concerned that scientists do not reveal work that could have commercial value and thus be protected by patent. To deal with this, some university scientists simply engage in avoidant behaviors including bypassing the commercialization office. Instead, they make their own judgments about the commercial potential and decide what to put in the public domain on that basis.

Lastly, scientists sometimes navigate the tension between openness and secrecy by engaging in what we have described as “sharing by not sharing”. For some, giving the appearance of sharing is important even if it is not genuine. In sharing by not sharing, they are able to save face, and meet the publication requirements of open research, while also being protective.

## 6 Conclusion

This research is part of a broader effort to understand the doing of synthetic biology research, in particular the tension between openness and secrecy in contemporary IP contexts. Our work showed that sharing is not generally driven by a negative view of the impact of patents on innovation in synthetic biology. This is a point of difference from the existing literature about open science and consortia in synthetic biology (see for example, Kahl and Molloy, 2018). Sharing is driven by cultural norms, scientific progress and strategic benefits to the sharer.

Further, our research has shown that the decision-making process around what to share and what to keep secret is more commonly influenced by the research context than core personal values about openness and ownership in biological research. That is, even though there is a culture of sharing in synthetic biology, scientists cannot get away from commercial realities associated with the roles they hold. Decisions not to share are necessarily influenced by the IP system. We have shown that where synthetic biologists were concerned about sharing, the barrier was typically the patent context (commercialization potential), or the funding context (commercial involvement and commercial contracts). Our research contributes an understanding of the way in which the “commercial value” of information is a slippery concept, and can be a complex judgement to make. We have shown that scientists use a distinction between fundamental and applied research in navigating this decision-making about commercial value and selective sharing.

The other key reason scientists chose not to share related to concerns about breach of trust and loss of scientific credit or priority and commercial opportunities. Our research contributes to the scholarly discussion of sharing in scientific research by providing a greater understanding of the importance of trust, reputation and reciprocal benefit. Scientists are managing a disjunct between a culture of openness and realities of being a working scientist. They decide what to do based on their research context, their scientific and career goals, the nature of the material or information being shared, and the nature of the recipient.

This research has focused on synthetic biology research in the Australian context. However, the synthetic biology community is global, and similar tensions between openness, secrecy, and commercialization arise in other countries. At present, there is a lack of empirical data about how these are being navigated in other parts of the world. It would be valuable for researchers to investigate the perspectives and practices of scientists and commercialization professionals in other countries. International comparisons would allow the impact of cultural variations and local conditions to be explored. This work could also facilitate international dialogue amongst synthetic biologists. We hope our contribution could be part of a comparative analysis when similar empirical work is done in other jurisdictions.

## Data Availability

The datasets presented in this article are not readily available due to confidentiality requirements of the ethical approval and participant consent. Requests to access the datasets are not applicable for this interview data.
